# Workload, job, and family satisfaction in dual-earning parents with adolescents: the mediating role of work-to-family conflict

**DOI:** 10.3389/fpsyg.2025.1529092

**Published:** 2025-03-26

**Authors:** Berta Schnettler, Andrés Concha-Salgado, Ligia Orellana, Mahia Saracostti, Katherine Beroiza, Héctor Poblete, Germán Lobos, Cristian Adasme-Berríos, María Lapo, Leonor Riquelme-Segura, José A. Sepúlveda, Karol Reutter, Enid Thomas

**Affiliations:** ^1^Facultad de Ciencias Agropecuarias y Medioambiente, Universidad de La Frontera, Temuco, Chile; ^2^Centro de Excelencia en Psicología Económica y del Consumo, Universidad de La Frontera, Temuco, Chile; ^3^Scientific and Technological Bioresource Nucleus (BIOREN-UFRO), Universidad de La Frontera, Temuco, Chile; ^4^Universidad Católica de Santiago de Guayaquil, Guayaquil, Ecuador; ^5^Departamento de Psicología, Universidad de La Frontera, Temuco, Chile; ^6^Departamento de Trabajo Social, Universidad de Chile, Santiago, Chile; ^7^Facultad de Economía y Negocios, Universidad de Talca, Talca, Chile; ^8^Departamento de Economía y Administración, Universidad Católica del Maule, Talca, Chile; ^9^Departamento de Trabajo Social, Universidad de La Frontera, Temuco, Chile; ^10^Doctorado en Ciencias Agroalimentarias y Medioambiente, Universidad de La Frontera, Temuco, Chile

**Keywords:** work-to-family conflict, dual-earning parents, adolescents, source attribution perspective, domain specificity model

## Abstract

**Introduction:**

The study examined the direct and indirect effects of parents’ workload, work-to-family conflict (WtoFC), job satisfaction, and family satisfaction among dual-earning parents and their adolescent children.

**Methods:**

A total of 516 dual-earning parents and one adolescent child were enlisted for the study using non-probabilistic sampling. Mothers and fathers completed assessments about workload, WtoFC, and the Overall Job Satisfaction Scale, while all three family members responded to the Satisfaction with Family Life Scale.

**Results:**

The data were analyzed using the mediation Actor-Partner Interdependence Model and structural equation modeling. The findings revealed a negative association between mothers’ workload and family satisfaction. Moreover, both parents’ workloads reduce adolescents’ family satisfaction. Both parents’ workload was positively associated with their WtoFC. Additionally, WtoFC was found to significantly mediate across intraindividual and interindividual domains, linking workload and job satisfaction in parents and workload and family satisfaction for parents and their adolescent children.

**Discussion:**

These empirical insights underscore the critical need to mitigate workload and WtoFC to enhance parental job satisfaction and family satisfaction of all family members. The study’s practical implications provide the audience with actionable insights that can be applied to workplace practices, empowering them to make informed decisions.

## 1 Introduction

The conservation of resources (COR) (Hobfoll, 1989) theory posits that individuals endeavor to attain and uphold their resources. Consequently, the strain may ensue when a disturbance in one’s professional or familial sphere leads to an incapacity to harmonize roles and safeguard resources in either domain, precipitating a decline in overall well-being (Yucel and Latshaw, 2020). Job demands appear to significantly impact as precursors to work-to-family conflict (WtoFC), which occurs when job responsibilities make it difficult for employees to meet their family obligations (Greenhaus and Beutell, 1985). Job demands encompass various job elements, including workload, which involves mental and physical exertion and can compromise the employees’ well-being (Landolfi et al., 2020). The workload combines job duties and other tasks that employees must complete within a specific timeframe (Janib et al., 2021).

Several studies have shown that workload increases employees’ WtoFC and decreases job and family satisfaction (e.g., Yucel and Latshaw, 2020; Landolfi et al., 2020; Basson and Rothmann, 2018; Shi et al., 2021). In this regard, it should be noted that there are two different perspectives on WtoFC. As suggested by Frone et al. (1992) the domain specificity model states that demands from the work domain impact the family domain. By contrast, the source attribution perspective posits that the influence of demands is pre-dominantly discernible within the sphere from which the demand emanates (Amstad et al., 2011, p. 155). According to research (Shockley and Singla, 2011), people encountering WtoFC may experience reduced performance in the domain affected by the conflict, which, in this case, is the family domain. However, they attribute this performance decline to the origin of the conflict, leading to decreased satisfaction in the work domain. The current study examined both approaches, yielding fresh perspectives on the influence of WtoFC on job and family satisfaction extending beyond the employee, whose understanding is still limited (Schnettler et al., 2024a).

Furthermore, in COR theory (Hobfoll, 1989), stress (i.e., WtoFC) depletes the employee’s personal resources like time and energy, which may also lead to decreased well-being among family members through crossover (Orellana et al., 2023), which denotes the transmission of stressors between individuals (Westman, 2001). However, most research on work–family conflict has centered on individual workers, leading to a gap in our understanding of its impact as it fails to consider the interconnected nature of conflict within couples and families (Burch, 2020; Orellana et al., 2021; Ratnaningsih and Idris, 2024). Therefore, to fill this gap, our research sought to assess the interrelationships among workload, WtoFC, and job and family satisfaction simultaneously in the employee, their partner, and their children, with the potential to significantly enhance our understanding of work–family conflict and its implications for family well-being.

In addition, most research on work–family conflict has been conducted in the United States of America and Europe, and more recently in Asian regions, mainly China, followed by Japan and South Korea (Ratnaningsih and Idris, 2024). For instance, there are studies that associate WtoFC and job satisfaction in Europe (Yucel and Latshaw, 2020) and China (Shi et al., 2021; Hong et al., 2021), studies that associate workload with WtoFC in Italy (Landolfi et al., 2020), Belgium (Kim et al., 2023), and the United States (Carlson et al., 2019), and studies that associated WtoFC with family satisfaction in Italy (Landolfi et al., 2020.) and the United States (Morr and Droser, 2020).

To bridge this divide, the research took place in a developing country in Latin America, specifically in Chile, where the recent passage of Law 21,561, commonly referred to as the 40-h Law, represents a significant shift in labor regulations. Formerly, the typical workweek in Chile spanned 45 h. On April 26, 2024, the first of several phases took effect, mandating a reduction to a 44-h workweek without any accompanying decrease in remuneration. Subsequently, by April 2026, the prescribed weekly working hours will be further reduced to 42, with full implementation of the 40-h workweek slated for 2028. The underlying intent of this reform is to elevate the well-being and overall quality of life for employees and their families (Dirección del Trabajo, 2023). Despite these noble aspirations, the practical realization of this initiative may inadvertently impose heightened work demands without achieving its intended primary objective. There has been no formal evaluation of the law’s effectiveness. However, analyses conducted before the implementation of Law 21,561 suggested a potential workload that merits consideration. For instance, the law changes the workday’s traditional “weekly” structure, allowing the 40-h workweek to be averaged over a four-week cycle rather than strictly distributed over seven days. In this case, an employer can decide that a worker may have a work week with 45 h, a week with 40 h, another with 35 h, and finally, one with 30 h. This flexible scheduling could lead to an increased workload during supposedly ‘shorter’ weeks (Universidad de Valparaíso, 2023).

Hence, building upon the COR theory, the domain specificity model, and the source attribution perspective, this study aimed to assess the direct and indirect individual and interindividual associations between workload, WtoFC, and job and family satisfaction in different-sex dual-earning parents with adolescents. Considering there is empirical support for both the domain specificity model and the source attribution perspective (e.g., Amstad et al., 2011, p. 153; Shockley and Singla, 2011), this study also compared the relationships between WtoFC and job satisfaction and between WtoFC and family satisfaction. Families with adolescents were chosen due to the recognition that, despite necessitating diminished parental supervision in comparison to younger counterparts (Matias and Recharte, 2021), teenagers are susceptible to experiencing parental conflict relating to the intersection of professional responsibilities and family obligations (Orellana et al., 2021).

### 1.1 Background and hypothesis

The COR theory suggests that individuals allocate resources to handle stressful situations like workload. When coping strategies are ineffective or require substantial resource investment, stress is likely to occur (Hobfoll, 2002), precipitating a decline in well-being (Yucel and Latshaw, 2020). In this regard, the effects of using up resources within the job domain can be understood through the examination of job satisfaction, which refers to the degree to which employees enjoy their work (Agho et al., 1992, p. 185) and plays a pivotal role in determining employee performance (Soomro et al., 2018). Similarly, assessing the impact of resource depletion in the family sphere may be done by analyzing satisfaction with family life (family satisfaction henceforth), constituting a deliberate, subjective cognitive assessment of an individual’s family life (Zabriskie and Ward, 2013).

Within the job domain, Basson and Rothmann (2018) found that increased workloads contribute to higher levels of pressure and discomfort among employees, ultimately leading to greater dissatisfaction with their organizations. Other recent studies showing a negative relationship between workload and job satisfaction have been conducted with a sample of educational support staff in Indonesia (Tentama et al., 2019), Australian nurses (Holland et al., 2019) university academic staff in Malaysia (Janib et al., 2021), female preschool teachers in China (Hong et al., 2021) and accounting professionals in Turkey (Baş and Güney, 2022). However, other authors have not found a direct association between these variables in a sample of university lecturers in Thailand (Kim et al., 2023).

The requirements of a job usually mean that workers must invest their resources, like time and effort, into their work. This can lead to insufficient time and energy for personal and family activities (Babic et al., 2020, p. 4). According to the COR theory (Hobfoll, 2002), the negative effect of workload on employees’ performance in their family life is caused by the depletion of resources due to the demanding efforts needed for the workload. Although the evidence in this regard is limited, in a study conducted in China (Huang et al., 2023), it was observed that an individual’s professional workload could impact their family life.

**Figure 1:**
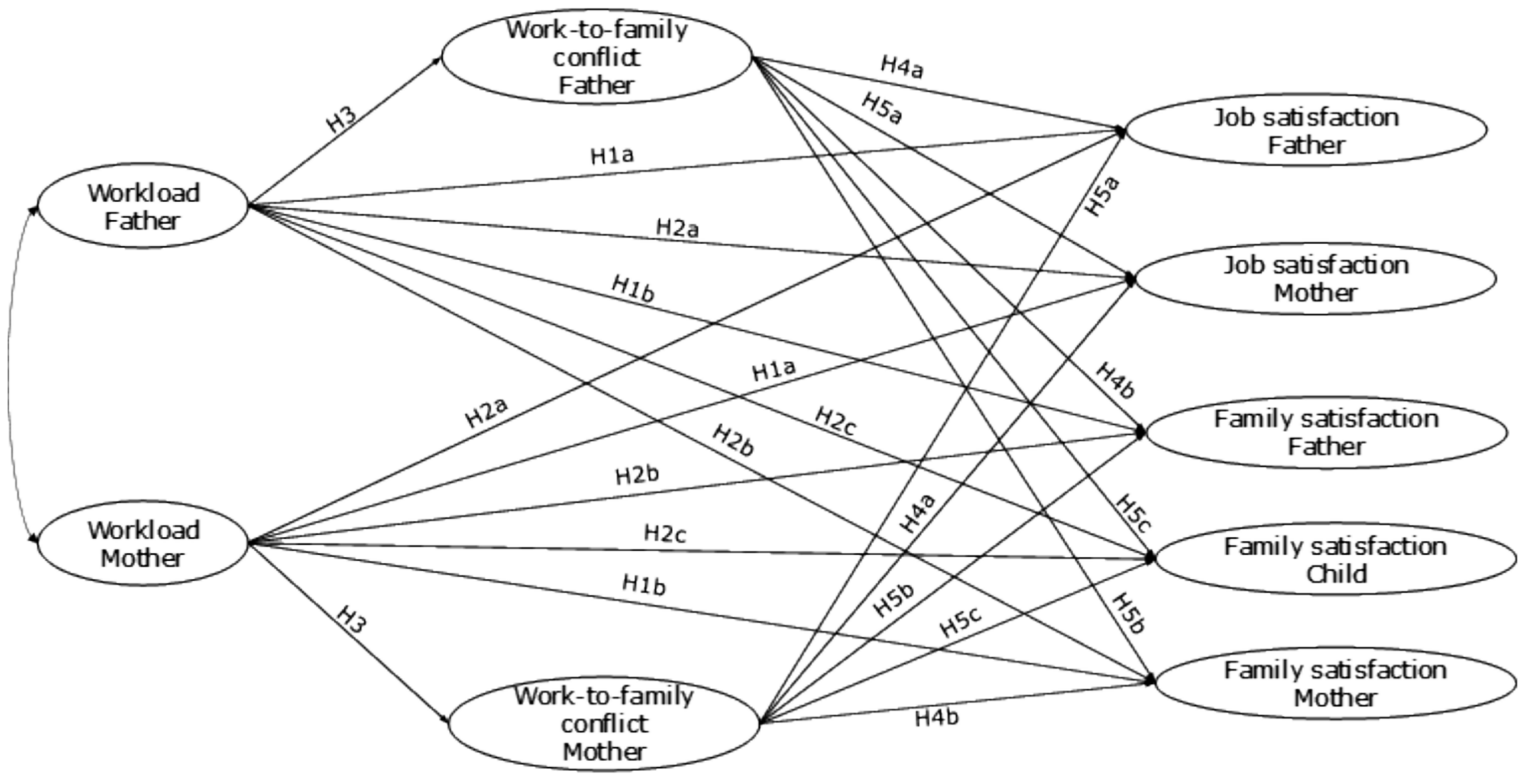
Conceptual model of the proposed actor and partner effects between workload, work-to-family conflict, job satisfaction, and family satisfaction in dual-earning parents with adolescent children. The conceptual path diagram did not include the indirect effects of WtoFC (H6 and H7) to prevent the figure from cluttering.

Therefore, based on the above theoretical and empirical background, the first hypothesis was posited that workload would have a negative direct relationship with job and family satisfaction at an individual level, as shown in Figure 1.

*H1*: Each parent’s workload is negatively related to their (a) job and (b) family satisfaction.

Regarding the crossover of stress (Westman, 2001), there is a paucity of studies involving workload and job and family satisfaction. However, some authors (Lavner and Clark, 2017; Lu et al., 2016) have posited that when one partner is overwhelmed by job demands such as workload, they may struggle to allocate enough time and energy to their family and marital aspects. Consequently, the burdened individual may struggle to contribute substantially to household tasks and other practical duties, escalating the family-related demands placed on the other partner. In response, the latter may allocate heightened resources to familial matters, depleting their capacity for professional engagements, consequently impacting their occupational performance (Soomro et al., 2018; Tentama et al., 2019; Ghislieri et al., 2021) and leading to a decline in job satisfaction (Schnettler et al., 2024b; Su and Jiang, 2023).

Furthermore, when one partner is overwhelmed by the workload, in addition to making a low contribution to domestic tasks, they can be unable to provide sufficient support to their partner due to their unavailability or emotional exhaustion or participate in enjoyable activities with their family (Lavner and Clark, 2017), decreasing the other partner’s and their children family satisfaction (e.g., Schnettler et al., 2024a; Orellana et al., 2023). Regarding this, in a longitudinal study involving a cohort of recently married couples in the United States, it was reported that partners of individuals with higher workloads experienced more declines in marital satisfaction (a component of family satisfaction) compared to those with lesser workloads (Lavner and Clark, 2017).

According to the theoretical and practical context, the second hypothesis proposes that one parent’s workload would have a negative correlation with the job and family satisfaction of the other parent and the family satisfaction of their teenage children (Figure 1).

*H2*: One parent’s workload is negatively related to (a) the other parent’s job satisfaction, (b) the other parent’s family satisfaction, and (c) the adolescents’ family satisfaction.

Resource depletion can potentially restrict the quantity and effectiveness of resources transitioning from the professional realm to the family domain, impacting the work-to-family dynamic. Workload, among the job demands, has been consistently emphasized in numerous studies as significantly associated with WtoFC (Babic et al., 2020, pp. 1–2). In this regard, the evidence shows that workload is positively related to WtoFC, such as studies conducted on Italian employees (Landolfi et al., 2020), employees from the health sector in Belgium (Babic et al., 2020), Chinese doctors (Shi et al., 2021), and nurses (Ekmekci et al., 2021) and accounting professionals from Turkey (Baş and Güney, 2022) Therefore, we posited the third hypothesis at an individual level (Figure 1).

*H3*: Each parent’s workload positively relates to their WtoFC.

Consistent with the source attribution perspective, a study found that increased WtoFC directly reduces job satisfaction among project-level construction professionals in Sri Lanka (Dodanwala and Shrestha, 2021). Similar results have been reported with a sample of dual-earning married couples in Germany (Yucel and Latshaw, 2020), female preschool teachers in China (Hong et al., 2021), Chinese doctors (Shi et al., 2021), university lecturers in Thailand (Kim et al., 2023), and with a sample of accounting professionals in Turkey (Baş and Güney, 2022).

From the domain specificity model perspective, research findings from Italy, the United States, Australia, Chile, and India show that employees who encounter WtoFC are inclined to report diminished levels of satisfaction in their familial roles (Landolfi et al., 2020; Burch, 2020; Orellana et al., 2021; Morr and Droser, 2020; Kalliath et al., 2017). In addition, the results of a recent meta-analysis on work-life balance (Matei et al., 2021) show a negative link between work-life conflict and family happiness, impacting both male and female partners.

Therefore, the fourth hypothesis posited a negative relationship between WtoFC and job and family satisfaction at an individual level in parents (Figure 1).

*H4*: Each parent’s WtoFC is negatively related to their (a) job and (b) family satisfaction.

The COR theory (Hobfoll, 1989) proposes that in situations involving the potential loss or actual depletion of resources, individuals can experience strain, negatively affecting both the employee and their family’s well-being through crossover (Hobfoll et al., 2018). Regarding the job domain, in Germany, the WtoFC experienced by husbands harmed their wives, ultimately resulting in a decline in job satisfaction of the wives (Yucel and Latshaw, 2020). Similarly, in Spain (Sanz-Vergel et al., 2024), it was discovered that when employees have high levels of WtoFC in the evening, their spouses are likely to remain mentally preoccupied with family-related thoughts the following day; this could then affect their work performance, leading to a negative impact on their job satisfaction (Schnettler et al., 2024b; Su and Jiang, 2023).

Regarding the family domain, some authors observed an inverse relationship between husbands’ WtoFC and wives’ family satisfaction and vice versa in dual-earning couples in China (Lu et al., 2016). Remarkably, another study discovered that when a wife experiences work–family conflict, it has a detrimental impact on her partner’s family-related well-being, while the work–family conflict in husbands negatively affects their wives’ family-related well-being (Matei et al., 2021).

Reimann et al. (2022) proposed that WtoFC adversely impacts the connection between parents and their children and parenting behaviors. For instance, the more a father experienced WtoFC, the less time he spent with his young children, as Kuo et al. (2018) demonstrated. During the pandemic, increased WtoFC is associated with a less favorable parent–child relationship in the Netherlands, particularly with young children (Verweij et al., 2021). Studies concerning parent–child relationships involving teenagers have found that children are affected by their parents’ work-life balance struggles, negatively impacting satisfaction in different life domains (Morr and Droser, 2020; Matias and Recharte, 2021; Schnettler et al., 2021). Additionally, some research has shown that how parents manage the transition between work and family life influences the home environment and subsequently affects children’s well-being (Leach et al., 2021). Other studies have reported indirect effects of parents’ WtoFC on children’s family satisfaction. In Chilean dual-earning families (Orellana et al., 2021), it was discovered that the struggle between parents’ work and family responsibilities was associated with how adolescents perceived their parents’ work, affecting their relationships. This perception, in turn, mediated how parents’ WtoFC conflict impacted the adolescents’ family satisfaction.

As a result, we formulated the fifth hypothesis that negatively associated one parent’s WtoFC with the other parent’s and their adolescent children’s well-being at an intraindividual level (Figure 1).

*H5*: One parent’s WtoFC is negatively associated with (a) the other parent’s job satisfaction, (b) the other parent’s family satisfaction, and (c) the adolescents’ family satisfaction.

Given that the core idea of the domain specificity model and the source attribution perspective is that WtoFC serves as a mediator between work and family domains (Frone et al., 1992; Shockley and Singla, 2011), we propose that WtoFC shows a mediating role between workload and job satisfaction and between workload and family satisfaction. In this regard, the research indicated that workload was linked to reduced job satisfaction due to its connection with WtoFC (Hong et al., 2021). According to recent findings, there is evidence to suggest that WtoFC serves as a mediating factor in the relationship between workload and job satisfaction (Baş and Güney, 2022, p. 354). Furthermore, the WtoFC of employees plays a mediating role, indirectly affecting both their family satisfaction and their spouses’ family satisfaction due to their job demands (Zhang et al., 2024). Furthermore, a recent study indicated that fathers’ family-to-work conflict mediates the link between their perceived family support and adolescents’ family satisfaction (Schnettler et al., 2024a). Thus, WtoFC may also play interindividual mediating roles between parents and their children. Therefore, based on this theoretical and empirical background, we posited the hypotheses sixth and seventh involving individual and interindividual associations.

*H6*: The WtoFC has a mediating role in the relationship between parents’ workload and job satisfaction.

*H7*: The WtoFC has a mediating role in the relationship between the parents’ workload and the three family members’ family satisfaction.

Lastly, some studies have simultaneously assessed the impact of WtoFC on family and job satisfaction. Thus, our last hypothesis compares the association related to the domain specificity model (WtoFC → family satisfaction) and the source attribution perspective (WtoFC → job satisfaction). In a meta-analysis (Amstad et al., 2011, pp. 153–154), it was found that WtoFC showed a significant association with both work-related and family-related outcomes. However, the link was more pronounced with work-related results than family-related results. Likewise, a meta-analytic study (Xu et al., 2018) reported a stronger correlation between WtoFC and job satisfaction than between WtoFC and family satisfaction. Similarly, in a cross-national meta-analytic study with 332 pre-pandemic studies, it was discovered that job and family satisfaction were negatively linked to WtoFC; the effect between WtoFC and job satisfaction was greater than the effect between WtoFC and family satisfaction (Allen et al., 2020). Some authors have reported that WtoFC significantly decreases job satisfaction, whereas the association between WtoFC and relationship satisfaction was insignificant in dual-earning couples (Yucel and Latshaw, 2020). More recently, during the pandemic, new research found that WtoFC negatively relates to job and family satisfaction in Chilean working mothers (Riquelme-Segura et al., 2022). However, these authors reported that the association between WtoFC and family satisfaction was stronger than the association between WtoFC and job satisfaction. Although this last study supports the domain specificity model over the source attribution perspective, the rest support the source attribution perspective over the domain specificity model. Therefore, we formulated the eighth hypothesis, which posits that the association between WtoFC and job satisfaction is significantly higher than between WtoFC and family satisfaction.

*H8*: The association between WtoFC and job satisfaction is significantly higher than the association between WtoFC and family satisfaction.

## 2 Methods

The inclusion criteria in the sample considered different-sex dual-earning parents (who were either married or living together) and at least one adolescent child aged between 10 and 15. Participants were recruited through a convenience non-probability sampling method, utilizing quotas corresponding to the community’s distribution of families based on Socioeconomic levels (high, medium, and low) in Temuco, Chile. This approach was designed to ensure a diverse socioeconomic level range sample. The sample consisted of 516 dual-earning families, 516 fathers, 516 mothers, and 516 adolescents, totaling 1,548 persons surveyed. This research is a component of a larger project examining the connections between work, family, and food-related demands in Chilean households (Schnettler et al., 2023a). The sample size was established by aiming for 10 participants for each item on the scales utilized in this study. This approach is informed by statistical simulation studies conducted by Gagne and Hancock (2006), who recommended including 7–12 participants per item, and suggestions from Kyriazos (2018) to work with 10 participants per item. Additionally, the sample size we obtained aligns with the suggestions of Ledermann et al. (2022), who indicate that a sample of 91 dyads is required to effectively identify mediated pathways among distinguishable dyads, while 249 dyads are necessary for assessing actor and partner effects. Our sample size exceeds these recommendations, as we aim to enhance the diversity of Chilean families.

Families were contacted through the teenagers’ schools and social networks. Trained interviewers explained the study’s aims and the questionnaire format to the parents and reassured them about the confidentiality and anonymity of the responses. Families agreeing to participate provided the email address of a family member, mainly the mother’s. The email address provided was used to send the surveys to each family member. Interviewers followed up by phone calls to assist with any queries and ensure the questionnaires were filled out. Data collection was conducted between June and November 2023.

On the first page of the online questionnaire, mothers and fathers were presented with a consent form, while adolescents were presented with an informed assent form. All family members agreed to participate in this research by checking a box. The completed questionnaires were each stored in separate databases on the QuestionPro platform (QuestionPro Inc). After filling out the three questionnaires, families were given a 15 USD bank transfer as compensation.

Forty families participated in a pilot test using the same recruitment method and data collection procedure, and no adjustments were necessary. The Ethics Committee of Universidad de La Frontera has approved the study (protocol number 035-23).

### 2.1 Measures

Mothers and fathers were asked to complete the following scales:

The workload scale (Bakker et al., 2004) consists of three items, such as “Do you have too much work to do?” Participants responded on a five-point scale ranging from 1 (never) to 5 (always). The study utilized the Spanish version of the scale, which was tested among Chilean workers (Schnettler et al., 2023a). Workload scores are calculated as the average of the three items, with higher scores indicating increased workload.

The Overall Job Satisfaction Scale (OJSS) (Agho et al., 1992) consists of six items, for example: “Most days I am enthusiastic about my job.” Respondents indicated their level of agreement with each statement using a five-point Likert scale; the scale ranges from 1 (strongly disagree) to 5 (strongly agree). The study utilized the validated Spanish version of the OJSS scale, demonstrating internal solid consistency in research involving dual-earning couples in Chile (Orellana et al., 2021; Schnettler et al., 2024b). The scores are calculated by summing the six items. The higher the scores on the OJSS, the greater the level of job satisfaction.

The researchers assessed WtoFC using a set of four questions adapted from Kinnunen et al. (2006, pp. 149–162). These questions aimed to understand the adverse impact of work on family life. For instance, one of the questions asked, “Does your job produce strain that makes it difficult to fulfil your family duties?.” Participants responded on a five-point scale ranging from 1 (never) to 5 (very often). The study utilized the Spanish version of the scale, validated with Chilean workers, and demonstrated solid internal consistency in samples of Chilean dual-earning parents (Orellana et al., 2021; Schnettler et al., 2021; Schnettler et al., 2023c). The scores are calculated by summing the four items. Higher scores on this measure indicate higher WtoFC.

The following scale was given to mothers, fathers, and adolescents to complete:

The Satisfaction with Family Life Scale (SWFaL) (Zabriskie and Ward, 2013) is a five-item scale adapted from the Satisfaction with Life Scale (Diener et al., 1985). The scale replaces the word “life” with “family life” in the five items, for example, “If I could live my family life over, I would change almost nothing.” Participants responded on a scale ranging from 1, indicating complete disagreement, to 6, indicating complete agreement. This study utilized the validated Spanish version of the scale, showing internal solid consistency in samples of Chilean adults and adolescents (Orellana et al., 2021; Schnettler et al., 2024b; Riquelme-Segura et al., 2022). The scores are calculated by summing the five items. Higher scores on the SWFaL indicate higher family satisfaction.

The surveys for the three family members included a question about their age; teenagers also had to state their gender. Parents were asked about their job type, weekly hours, and work arrangements (remote, in-person, hybrid). Mothers were queried about the size of their family and the number of children. The family’s socioeconomic status (SES) was calculated based on the household’s total income and size and the education and occupation of the partner who contributes the most income to the household (Agencia de Investigación para el Mercado, 2024).

### 2.2 Data analysis

The descriptive analysis was conducted using SPSS v.23. The Workload scale has not been utilized in dyadic analysis in the Spanish language. To address this gap, a dyadic confirmatory factor analysis (CFA), consistent with the methodology outlined by Claxton et al. (2015), was undertaken to scrutinize its latent structure and psychometric properties. Internal consistency was evaluated using the Omega coefficient (McDonald, 1970). Convergent validity was appraised by scrutinizing the standardized factor loadings of the scale (preferably >0.5), in addition to their statistical significance and average variance extracted (AVE, values >0.5) (McDonald, 1970). These same measures were applied to the remaining scales utilized in this study.

To assess Hypotheses 1–8, the mediation Actor-Partner Interdependence Model (APIM) and structural equation modeling (SEM) were used following the approach outlined by Kenny et al. (2006). This study examined the connections between variables for individual family members (actor effects) and between family members (interindividual, crossover, or partner effects). Fathers and mothers were considered actors and partners, while teenagers were only considered partners. The analysis examined the associations between parents’ workload, WtoFC, job satisfaction (OJSS), and family members’ family satisfaction (SWFaL).

The APIM helps manage the interconnectedness among family members. The study included correlations between each parent’s workload to account for the association of this variable between mothers and fathers. According to the methodology outlined by Kenny et al. (2006), the APIM also incorporates correlations between the residual errors of the dependent variables, specifically the parental dependent variables (WtoFC and OJSS) and those of the three family members (SWFaL). According to these authors, these correlations help account for additional sources of interdependence among the members of each dyad.

Additionally, the analysis included variables that directly impact the dependent variables for both mothers and fathers (WtoFC and OJSS) as well as the family satisfaction of all three family members (SWFaL). This was done to statistically adjust for external factors that could influence the relationships among the key variables in the model. The variables were the ages of the three family members, the employment status of both parents, their working hours and workplace, family socioeconomic status, and the number of children. Previous studies have indicated a positive correlation between age and job satisfaction (Dobrow Riza et al., 2018), prompting the inclusion of parents’ age as a control variable. The age of adolescents was also considered, as research suggests that older adolescents tend to have a better understanding of how their parents’ jobs impact their lives compared to younger ones (Matias and Recharte, 2021). Additionally, the type of employment was factored in, given that self-employed individuals generally report higher job satisfaction (Janicijevic and Paunović, 2019; Schnettler et al., 2023b). This satisfaction is linked to aspects such as workplace autonomy, flexibility, a sense of personal responsibility, safe work conditions, and a supportive work environment (Janicijevic and Paunović, 2019). Furthermore, studies show that self-employed mothers tend to experience higher levels of family satisfaction than their employed counterparts (Orellana et al., 2021). Working hours were also considered since part-time workers often have lower monthly incomes than full-time employees, which can influence job and family satisfaction (Orellana et al., 2023). Finally, family SES was included as another control variable, as employees from lower SES backgrounds tend to report decreased job satisfaction (Li et al., 2020; Pieh et al., 2020).

Mplus 8.11 was used to conduct the CFA and SEM, with factor loadings and structural parameters estimated using the Unweighted Least Squares Mean and Variance adjusted (ULSMV) method. The polychoric correlation matrix was used because the items were measured on an ordinal scale. The adequacy of the CFA and SEM models was evaluated using the Tucker-Lewis index (TLI) and the comparative fit index (CFI). A value above 0.95 indicates a good fit, while a value above 0.90 suggests an acceptable fit. The root mean square error of approximation (RMSEA) was also employed, with values below 0.06 indicating a good fit and values below 0.08 denoting an acceptable fit (Hu and Bentler, 1999).

Following the methodology suggested by Lau and Cheung (2012), SEM assessed WtoFC’s mediating role (Hypotheses 7 and 8), utilizing a bias-corrected (BC) bootstrap confidence interval with 1,000 samples. Intervals that exclude zero reveal an indication of a mediating role.

Differences between the association of WtoFC and job satisfaction (source attribution perspective) and WtoFC and family satisfaction (domain specificity model) path coefficients were tested via SEM at individual and interindividual levels for each parent, from one parent to the other, and from each parent to the adolescent (Hypothesis 9).

## 3 Results

Table 1 presents the sociodemographic features of the sample. There were no missing data. The sample consisted of 516 families, encompassing mothers, fathers, and teenagers, totaling 1,548 family members who were surveyed. Most households demonstrated a middle socio-economic status and generally included an average of four family members with two children. Most parents were employed, with fathers working more hours per week than mothers. Additionally, most fathers and mothers worked on-site.

**Table 1:** Sociodemographic features of the sample.

Characteristic	Total sample
Age [Mean (*SD*)]	
Mother	37.1 (6.9)
Father	39.8 (7.8)
Adolescent	12.2 (1.7)
Adolescents’ gender (%)	
Male	51.0
Female	49.0
Number of family members [Mean (*SD*)]	4.2 (0.9)
Number of children [Mean (*SD*)]	2.1 (0.9)
Socioeconomic status (%)	
High	4.1
Middle	85.1
Low	10.9
Mothers’ type of employment (%)	
Employee	70.3
Self-employed	29.7
Fathers’ type of employment (%)	
Employee	73.6
Self-employed	26.4
Working hours [Mean (SD)]	
Mothers	33.6 (14.9)
Fathers	42.5 (13.0)
Mothers’ place of working (%)	
Remote	3.1
In-person	86.4
Mixed	10.5
Fathers’ place of working (%)	
Remote	1.6
In-person	92.6
Mixed	5.8

Sample characteristics (*n* = 516).

Table 2 depicts the average scores and correlations for parents’ workload, work-to-family conflict (WtoFC), job satisfaction (OJSS), and the three family members’ family satisfaction (SWFaL). Most connections showed statistical significance and corresponded with the anticipated pattern, except those between mothers’ workload and fathers’ OJSS and SWFaL. Also, between fathers’ workload and mothers’ WtoFC, their and the mothers’ OJSS and the three family members SWFaL. Fathers had significantly higher workload scores than mothers (*t* = −4.271, *p* < 0.001), but there were no significant differences between fathers and mothers in their WtoFC (*t* = 0.115, *p* = 0.908) and OJSS (*t* = 0.496, *p* = 0.620) scores. Mothers scored significantly lower than fathers and adolescents in SWFaL (*F* = 8.312, *p* < 0.001), while fathers did not differ from adolescents.

**Table 2:** Descriptive statistics and correlations for workload, work-to-family conflict (WtoFC), job satisfaction (OJSS), and satisfaction with family life (SWFaL) in different-sex dual-earning parents with adolescent children (*n* = 516).

	M (SD)	Correlations								
		1	2	3	4	5	6	7	8	9
Mothers’ workload	3.5 (0.9)	1	0.402***	0.339***	0.158***	−0.109*	−0.040	−0.154***	0.012	−0.079
Fathers’ workload	3.7 (0.9)		1	0.085	0.261***	−0.049	−0.055	−0.020	0.036	0.022
Mothers’ WtoFC	10.7 (4.2)			1	0.265***	−0.230***	−0.090*	−0.197***	−0.133**	0.109*
Fathers’ WtoFC	10.7 (4.4)				1	−0.135**	−0.166***	−0.161***	−0.131**	0.144**
Mothers’ OJSS	21.5 (4.5)					1	0.279***	0.237***	0.200***	0.138**
Fathers’ OJSS	21.3 (4.4)						1	0.129**	0.260**	0.091*
Mothers’ SWFaL	23.7 (44.6)							1	0.521***	0.463***
Fathers’ SWFaL	24.5 (4.2)								1	0.428***
Adolescents’ SWFaL	24.8 (4.5)									1

*p < 0.05, **p < 0.01, ***p < 0.001.

### 3.1 Psychometric properties of the workload scale

Results for the dyadic CFA indicated that the Workload scale measurement model fits well with the data for mothers and fathers (RMSEA = 0.075; CFI = 0.992; TLI = 0.975). The results indicated that the scale demonstrated high reliability, with Omega coefficients of 0.88 for mothers and 0.89 for fathers. Additionally, all factor loadings were found to be statistically significant (*p* < 0.001), and their values support convergent validity (mothers’ range = 0.746–0.979, fathers’ range = 0.688–0.928). The AVE was 0.72 for mothers and fathers.

### 3.2 APIM results: testing individual and interindividual hypotheses

All the standardized factor loadings of the scales used were statistically significant (*p* < 0.001) and exceeded 0.50, thus supporting convergent validity. The AVE values were good (mothers’ workload = 0.71, fathers’ workload = 0.73, mothers’ WtoFC = 0.74, fathers’ WtoFC = 0.77, mothers’ OJSS = 0.59, fathers’ OJSS = 0.59, mothers’ SWFaL = 0.74, fathers’ SWFaL = 0.69, adolescents’ SWFaL = 0.73). The omega coefficients indicated a good level of reliability for all the scales (mothers’ workload = 0.87, fathers’ workload = 0.89, mothers’ WtoFC = 0.92, fathers’ WtoFC = 0.93, mothers’ OJSS = 0.89, fathers’ OJSS = 0.89, mothers’ SWFaL = 0.94, fathers’ SWFaL = 0.93, adolescents’ SWFaL = 0.94).

**Figure 2:**
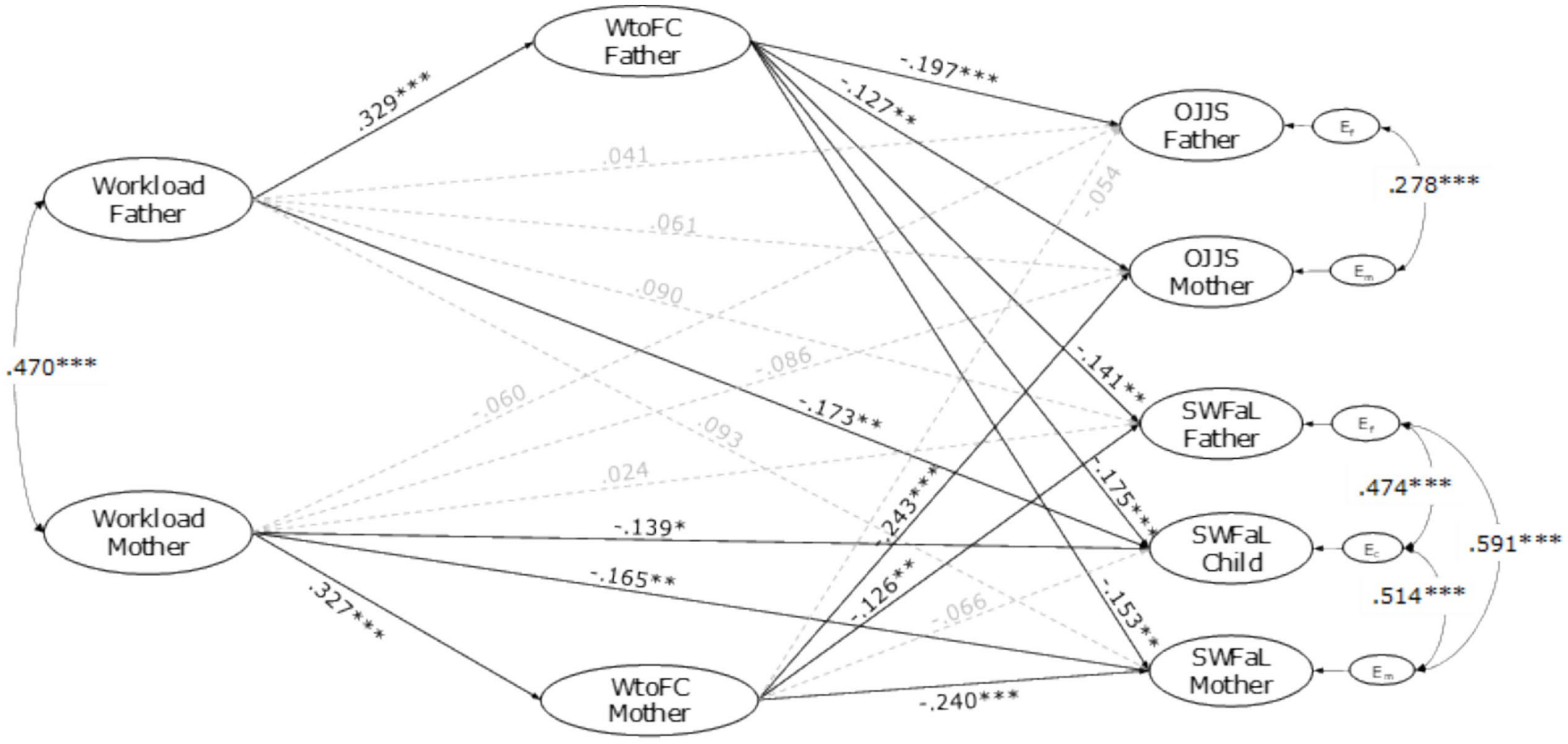
Actor-partner interdependence model of the effect of workload, WtoFC (WtoFC), job satisfaction (OJSS), and family satisfaction (SWFaL) in dual-earning parents with adolescent children. Em, Ec, and Ef: residual errors on OJSS and SWFaL for mothers, adolescent children, and fathers, respectively. *p < 0.05, **p < 0.01, ***p < 0.001.

The results obtained from estimating the structural model are depicted in Figure 2.

The APIM model exploring the link between the mother’s and father’s workload, work-to-family conflict (WtoFC), and job satisfaction (OJJS), and the three family members’ family satisfaction (SWFaL) had a good fit with the data (CFI = 0.956; TLI = 0.950; RMSEA = 0.022). Significant correlations were found between parents’ workload (*r* = 0.470, *p* < 0.001). Significant correlations were also found between the residual errors of mothers’ and fathers’ WtoFC (*r* = 0.288, *p* < 0.001), OJJS (*r* = 0.278, *p* < 0.001) and SWFaL (*r* = 0.591, *p* < 0.001), between mothers’ and adolescents’ SWFaL (*r* = 0.514, *p* < 0.001), and between fathers’ and adolescents’ SWFaL (*r* = 0.474, *p* < 0.001).

The family SES, the adolescents’ ages, the number of children, the mothers’ and fathers’ type of employment, and the mothers’ number of working hours per week affected the model significantly as control variables. The family SES positively affected the fathers’ OJSS (*γ* = 0.105, *p* = 0.030) and the mothers’ SWFaL (*γ* = 0.106, *p* = 0.025). The adolescents’ age negatively affected their SWFaL (*γ* = −0.195, *p* < 0.001). The number of children negatively affected the fathers’ SWFaL (*γ* = −0.101, *p* = 0.037). The fathers’ type of employment (employee vs. independent) positively affected their OJSS (*γ* =0.154, *p* = 0.001). A similar result was obtained in mothers (*γ* =0.116, *p* = 0.019). Lastly, the mothers’ number of working hours per week negatively affected their SWFaL (*γ* = −0.137, *p* = 0.006).

As Figure 2 depicted, the path coefficients show that fathers’ (*γ* = 0.041 *p* = 0.447) and mothers’ (*γ* = −0.086, *p* = 0.093) workload was not significantly associated with their OJSS. Whereas fathers’ workload was not significantly associated with their SWFaL (*γ* = 0.090, *p* = 0.087), mothers’ workload was negatively associated with their SWFaL (*γ* = −0.165, *p* = 0.002). These results did not support H1a for actor effects, whereas they supported H1b only for mothers.

Fathers’ workload was not significantly associated with the mothers’ OJSS (*γ* = 0.061, *p* = 0.299), and mothers’ workload was not significantly associated with the fathers’ OJSS (*γ* = −0.060, *p* = 0.240). Similarly, neither the fathers’ workload was significantly related to the mothers’ SWFaL (*γ* = 0.093, *p* = 0.080) nor the mothers’ workload was significantly related to the fathers’ SWFaL (*γ* = 0.090, *p* = 0.087). The father’s (*γ* = −0.173, *p* = 0.002) and the mothers’ (*γ* = −0.139, *p* = 0.018) workload was negatively associated with the adolescents’ SWFaL. These findings did not support H2a and H2b for partner effects, but they supported H2c for fathers and mothers.

Fathers’ (*γ* = 0.329, *p* < 0.001) and mothers’ (*γ* = 0.327, *p* < 0.001) workload was positively associated with their own WtoFC; thus, H3 was supported for actor effects.

Fathers’ (*γ* = −0.197, *p* < 0.001) and mothers’ (*γ* = −0.240, *p* < 0.001) WtoFC was negatively related to their OJSS. Likewise, fathers’ (*γ* = −0.141, *p* = 0.003) and mothers’ (*γ* = −0.149, *p* = 0.002) WtoFC was negatively related to their SWFaL. These findings supported H4a and H4b for actor effects.

Whereas fathers’ WtoFC was negatively related to the mothers’ OJSS (*γ* = −0.127, *p* = 0.006), mothers’ WtoFC was not associated with the fathers’ OJSS (*γ* = −0.054, *p* = 0.260). The fathers’ WtoFC was negatively associated with the mothers’ SWFaL (*γ* = −0.153, *p* = 0.002), and the mothers’ WtoFC was negatively associated with the fathers’ SWFaL (*γ* = −0.126, *p* = 0.014). Whereas the fathers’ WtoFC was negatively associated with the adolescent’s SWFaL (*γ* = −0.175, *p* < 0.001), the mother’s WtoFC was not significantly associated with the adolescents’ SWFaL (*γ* = −0.066, *p* = 0.190). These findings supported H5a for fathers and H5b for partner effects, while they supported H5c only for fathers.

### 3.3 The mediating role of the work-to-family conflict

The study also examined how parents’ WtoFC mediates the relationship between parents’ workload and job satisfaction (H6) and between parents’ workload and family members’ family satisfaction (H7). It was found that the father’s WtoFC is a mediator in the connection between their workload and job satisfaction, as evidenced by a significant indirect effect (Table 3). A similar result was found in the relationship between the fathers’ workload and the mothers’ OJSS. The mediating role of mothers’ WtoFC was found to apply solely to the correlation between their workload and job satisfaction. These findings partially supported H6.

**Table 3:** Bias-corrected confidence intervals of specific mediation effects of parent’s work-to-family conflict (WtoFC).

Specific indirect effects	Estimate	Lower 2.5%	Upper 2.5%	*p*-value
Fathers’ workload → Fathers’ WtoFC → Fathers’ OJSS	−0.065	−0.099	−0.031	0.001**
Mothers’ workload → Mothers’ WtoFC → Fathers’ OJSS	−0.018	−0.048	0.013	0.258
Fathers’ workload → Fathers’ WtoFC → Mothers’ OJSS	−0.042	−0.074	−0.010	0.010*
Mothers’ workload → Mothers’ WtoFC → Mothers’ OJSS	−0.078	−0.114	−0.042	<0.001***
Fathers’ workload → Fathers’ WtoFC → Fathers’ SWFaL	−0.046	−0.081	−0.012	0.008**
Mothers’ workload → Mothers’ WtoFC → Fathers’ SWFaL	−0.041	−0.076	−0.007	0.020*
Mothers’ workload → Mothers’ WtoFC → Adolescents’ SWFaL	−0.022	−0.055	0.011	0.198
Fathers’ workload → Fathers’ WtoFC → Adolescents’ SWFaL	−0.058	−0.096	−0.020	0.003**
Fathers’ workload → Fathers’ WtoFC → Mothers’ SWFaL	−0.050	−0.086	−0.009	0.006**
Mothers’ workload → Mothers’ WtoFC → Mothers’ SWFaL	−0.049	−0.080	−0.018	0.002**

WL, Workload; OJSS, Job satisfaction; SWFaL, Family satisfaction. *p < 0.05, **p < 0.01, ***p < 0.001.

The role of the father’s WtoFC as a mediator in the relationship between their workload and family satisfaction was supported. The fathers’ WtoFC also mediates between their workload and the mothers’ and adolescents’ family satisfaction. The mothers’ WtoFC mediates between their workload and the fathers’ family satisfaction and between the fathers’ workload and the mothers’ family satisfaction. These results partially supported H7.

### 3.4 Testing differences between source attribution perspective and domain specificity model paths

In Table 4, the link between WtoFC and job satisfaction was not significantly different from the link between WtoFC and family satisfaction for both mothers and fathers on an individual level (*p* > 0.05). Similar results were found at the level of comparison between one parent’s WtoFC and the other parent’s family and job satisfaction and between each parent’s WtoFC and the family satisfaction of adolescents. Therefore, hypothesis 8 was not supported.

**Table 4:** Difference between the source attribution perspective and the domain specificity model paths.

Effects differences	*p*-value
(Mothers’ WtoFC → Mother’s OJSS) – (Mothers’ WtoFC→ Mothers’ SWFaL)	0.316
(Fathers’ WtoFC → Father’s OJSS) – (Fathers’ WtoFC→ Mothers’ SWFaL)	0.129
(Mothers’ WtoFC → Father’s OJSS) – (Mothers’ WtoFC→ Fathers’ SWFaL)	0.253
(Fathers’ WtoFC → Mother’s OJSS) – (Fathers’ WtoFC→ Mothers’ SWFaL)	0.685
(Fathers’ WtoFC → Adolescents’ SWFaL) – (Mothers’ WtoFC→ Adolescents’ SWFaL)	0.188

^*^WtoFC, Work-to-family conflict; OJSS, Job satisfaction; SWFaL, Family satisfaction.

## 4 Discussion

Drawing on the COR theory, the domain specificity model, and the source attribution perspective, this is the first study that has simultaneously assessed individual and interindividual interrelations between workload, WtoFC, and job and family satisfaction in different-sex dual-earning parents with adolescents. Using the mediation APIM, we found that mothers’ workload directly decreases their family satisfaction but not their job satisfaction. By contrast, fathers’ workload showed a null direct association with their job and family satisfaction. However, both parents’ workload was negatively linked to the teenagers’ family satisfaction. Parents’ workload was positively linked to their WtoFC, which in turn negatively influenced their family and job satisfaction and the adolescents’ family satisfaction with differences associated with the parent’s gender. These findings are discussed below, whereas lastly, we discussed the comparison between the domain specificity model and the source attribution perspective.

### 4.1 Workload, domain satisfaction, and work-to-family conflict

We expected that the loss of resources due to workload would decrease both parents’ job satisfaction (H1a) and family satisfaction (H1b). However, contrary to evidence showing an adverse direct association between workload and job satisfaction in diverse economic sectors and countries (Janib et al., 2021; Basson and Rothmann, 2018; Tentama et al., 2019; Baş and Güney, 2022), Hypothesis 1a was not supported. Namely, workload was not directly associated with job satisfaction in the sample under study, regardless of the parent’s gender. The difference between our results and those of Janib et al. (2021) and Baş and Güney (2022) may be explained by the fact that these authors did not incorporate a mediating variable between workload and job satisfaction. Nevertheless, the rest of the authors used a mediating variable in their models. In these cases, one possible explanation may be that the relationship between workload and job satisfaction is culture-sensitive. By contrast, our findings align with a recent study reporting that workload was negatively related to job satisfaction only via job stress (Kim et al., 2023). Therefore, although our results did not support a direct association between workload and job satisfaction, they support indirect associations, as discussed below.

Hypothesis 1b was supported only for mothers. This means that mothers’ workload reduces their family satisfaction, not fathers. This result in mothers is in line with the COR theory, which posits that the adverse impact of workload on employees’ family satisfaction can be ascribed to the depletion of resources resulting from the demanding efforts necessitated by the workload (Hobfoll, 2002; Babic et al., 2020, p. 4) and with recent evidence in China (Huang et al., 2023). Therefore, our findings on mothers expand the knowledge in another culture. The null association between fathers’ workload and family satisfaction aligns with Lavner and Clark's (2017) suggestion that employees may be able to adapt to their workload. These findings may stem from the enduring traditional gender roles in Chile, in which mothers still primarily take on family caregiving and household chores responsibilities (Schnettler et al., 2024b). Hence, it is likely that mothers’ workload negatively affects their performance in tasks involving their family, making them incapable of meeting their family’s expectations, therefore negatively affecting their family satisfaction. On the other hand, fathers usually act as the primary providers for the family (Schnettler et al., 2024b). Hence, they most likely view having a workload as a regular aspect of their gender role, which does not impact their family satisfaction.

We also hypothesized that one parent’s workload would be negatively associated with the other parent’s job satisfaction (H2a), the other parent’s family satisfaction (H2b), and the teenagers’ family satisfaction (H2c). However, H2a and H2b were not supported; one parent’s workload did not cross over to the other parent’s job and family satisfaction, regardless of the parent’s gender. Therefore, our findings (H2a) contradict earlier studies that suggest one partner’s workload can negatively impact the other partner’s job satisfaction (Ghislieri et al., 2021; Lu et al., 2016; Lavner and Clark, 2017; Schnettler et al., 2024b; Soomro et al., 2018; Su and Jiang, 2023; Tentama et al., 2019). One possible explanation for our findings may be related to the composition of the sample, which includes both parents and one adolescent, as well as the number of variables incorporated in the model that also account for family satisfaction. In this context, our results contradict a previous longitudinal study that reported partners of individuals with higher workloads experienced greater declines in marital satisfaction compared to those with lesser workloads among recently married couples (Lavner and Clark, 2017). Therefore, the difference between our results and those of Lavner and Clark (2017) may be associated with the different stages of life of the families in both samples or with the cross-sectional nature of the sample of the present study.

However, the boundary theory provides insight into the absence of the crossover effects. According to this theory, individuals navigate the boundaries between their professional and personal lives by employing either segmentation or integration strategies (Ashforth et al., 2000, pp. 476–477). For instance, employees who integrate their professional and personal lives tend to combine and interconnect elements from both domains by removing barriers between them. Conversely, those who opt for segmentation designate clear boundaries to prevent the spillover of elements from one domain to the other. Therefore, our findings suggest that each parent likely attempts to separate their professional and family lives, which helps reduce the direct impact of their workload on the other parent’s job and family satisfaction.

By contrast, Hypothesis 2c was supported for mothers and fathers, showing that both parents’ workloads directly crossed over to the adolescents, decreasing their family satisfaction. This shows that despite the attempts made by parents contending with high workloads to delineate boundaries between their professional and familial spheres, they may exhibit increased negative interactions with their adolescent children and struggle to offer substantial support due to constraints in availability or emotional reserves. Consequently, their ability to partake in activities with their children may be compromised, resulting in diminished adolescent family satisfaction. It is important to highlight that this finding broadens our understanding of how employees’ workloads can negatively affect the well-being of their adolescent children.

Workload, as a mechanism of resource depletion, has been identified as one of the primary precursors of WtoFC (Babic et al., 2020, pp. 4–5). Our results support Hypothesis 3 for mothers and fathers; that is, their workload was directly and positively related to their levels of WtoFC. The results are consistent with previous research conducted on an individual basis with employees from different sectors and cultures (Landolfi et al., 2020; Shi et al., 2021; Baş and Güney, 2022; Babic et al., 2020; Ekmekci et al., 2021).

### 4.2 Work-to-family conflict, job satisfaction, and family satisfaction

Drawing on the source attribution perspective (Amstad et al., 2011, p. 155; Shockley and Singla, 2011) and the domain specificity model perspective (Frone et al., 1992), we expected that each parent’s WtoFC would be negatively associated with their job (H4a) and family satisfaction (H4b). Hypothesis 4a was supported for mothers and fathers, which aligns with the source attribution perspective and with evidence showing a negative association between WtoFC and job satisfaction at an individual level (Yucel and Latshaw, 2020; Shi et al., 2021; Hong et al., 2021; Kim et al., 2023; Dodanwala and Shrestha, 2021). Likewise, H4b was also supported for mothers and fathers, which is consistent with the domain specificity model perspective and with previous studies showing a negative association between WtoFC and family satisfaction at an individual level (Landolfi et al., 2020; Burch, 2020; Orellana et al., 2021; Kim et al., 2023; Morr and Droser, 2020; Matei et al., 2021).

The COR theory suggests that individuals may experience strain and adverse effects on their well-being and their families’ well-being when facing the risk of resource loss or actual loss. This can occur through crossover, as Hobfoll et al. (2018) explain. On this basis, we hypothesized that one parent’s WtoFC would be negatively related to the other parent’s job satisfaction (H5a), the other parent’s family satisfaction (H5b), and the adolescents’ family satisfaction (H5c). Hypothesis 5a was supported only for fathers; fathers’ WtoFC crossed over to the mothers, decreasing their job satisfaction but not vice versa. These results align with the findings of Yucel and Latshaw (2020) in Germany. In this regard, it has been reported that in couples who share family and household responsibilities, one partner may experience potential or actual resource loss when the other experiences WtoFC (Lu et al., 2016; Lavner and Clark, 2017). Therefore, our findings indicate that when fathers face WtoFC and cannot manage household responsibilities, mothers must invest their time and energy in handling these tasks. As a result, their performance at work may suffer, either because they had to redirect resources initially meant for work toward family responsibilities (Lu et al., 2016; Lavner and Clark, 2017) or due to being mentally preoccupied with family-related issues (Sanz-Vergel et al., 2024), ultimately leading to decreased job satisfaction (Schnettler et al., 2024b; Su and Jiang, 2023) through crossover. As Yucel and Latshaw (2020) explained, this asymmetrical partner effect may be due to women exhibiting a higher susceptibility to influence from their partners than men. This disparity is attributed to the socio-cultural conditioning of women, rendering them more attuned to their partner’s needs and preferences than their male counterparts (Westman, 2001).

By contrast, Hypothesis 5b was supported for mothers and fathers; that is, mothers’ WtoFC crossed over to the fathers, decreasing their family satisfaction and vice versa. This symmetrical partner effect is in line with the COR theory, which suggests that when people face challenges in meeting their own needs due to their partner’s WtoFC, they perceive a risk of losing essential resources. This perception, in turn, leads to decreased family satisfaction (Lu et al., 2016).

Similarly to Hypothesis 5a, H5c was supported only for fathers; that is, only fathers’ WtoFC crossed over to the adolescents, decreasing their family satisfaction. This finding is in line with earlier studies, both with samples of younger children and adolescents (Kuo et al., 2018; Leach et al., 2021; Matias and Recharte, 2021; Morr and Droser, 2020; Orellana et al., 2021; Reimann et al., 2022; Schnettler et al., 2021; Verweij et al., 2021) that highlighted that parents’ WtoFC negatively affects the parent–child relationship, which in turn indirectly decreases children’s satisfaction in different life domains. Nevertheless, our findings expand on the knowledge showing that fathers’ WtoFC directly influences the adolescents’ family satisfaction.

The unanticipated outcome revealing gender disparities was the absence of a crossover effect from mothers’ WtoFC to the teenagers’ family satisfaction. This finding is noteworthy, given that mothers and fathers reported comparable levels of WtoFC in this sample. The finding above contrasts with prior research, indicating that the impact of WtoFC is generally more pronounced among mothers, who are traditionally regarded as primary caregivers with whom their children have closer interactions (Matias and Recharte, 2021). A study by Caro et al. (2017) revealed that Chilean teenagers’ perceptions of their parents’ work-family tension and demands disproportionately affected mothers compared to fathers. The adolescents perceived their mother’s employment as a significant source of conflict and emotional strain, which interfered with other aspects of their lives, resulting in their mothers experiencing chronic fatigue and distress. Researchers found that mothers seem to separate family-related stress from work during the pandemic more than fathers do (Schnettler et al., 2024a). Given this finding, it is plausible that mothers may separate their WtoFC from their children’s interactions. As a result, the adverse effects of mothers’ WtoFC on their adolescent children’s family satisfaction may be less pronounced. However, another feasible explanation for gender differences may be related to fathers exhibiting high levels of WtoFC who tend to adopt a less assertive communication style with their children. This is attributed to the reduced availability of personal resources, which are predominantly allocated to meeting professional obligations (Orellana et al., 2021).

An alternative interpretation of the findings presented in Hypotheses 4 and 5 pertains to the relationship between WtoFC and psychological distress and the implications of psychological distress for job and family satisfaction. Previous studies have identified WtoFC as a substantial stressor (Poms et al., 2016) that adversely affects employees’ mental health (Carvalho et al., 2018). Individuals experiencing elevated levels of WtoFC may endure heightened tension and threats to their mental resources, ultimately leading to increased psychological distress (Abdou et al., 2024, p. 3). This distress not only impacts the individual but also extends to family dynamics (Davis et al., 2017; Yucel, 2017), which may further affect the emotional well-being of their children through crossover effects (Lam et al., 2018).

The COR theory elucidates a loss spiral whereby psychological distress can threaten or deplete personal resources, such as energy and skills (Hobfoll et al., 2018), which may negatively influence job performance and diminish job satisfaction. Evidence suggests that psychological distress can impair employees’ capacity to manage job demands, significantly reducing their performance. Additionally, psychological distress has been identified as a critical factor associated with low job satisfaction among both male and female employees (Ghawadra et al., 2019; Simard et al., 2022). Although research on the crossover effects of one partner’s psychological distress on the other’s job satisfaction remains limited, a recent study indicated that fathers’ psychological distress negatively affected mothers’ job satisfaction. In contrast, the reverse effect was not documented (Schnettler et al., 2024b).

Furthermore, psychological distress has been shown to correlate negatively with family satisfaction among adults (Karani et al., 2022; Lu et al., 2018) and adolescents (Saleem et al., 2021; Wong et al., 2022) at the individual level. Certain studies posit that this resource depletion can extend to family members (Hobfoll et al., 2018). For instance, the mental health of an individual can influence the well-being of their partner (Orellana et al., 2022; Schnettler et al., 2019), while depressive symptoms can detrimentally affect family interactions (Lu et al., 2018). Research involving different-sex dual-earning couples has revealed that depressive symptoms correlate negatively with both their own and their partner’s family satisfaction (Orellana et al., 2022; Schnettler et al., 2019).

### 4.3 The mediating role of work-to-family conflict

We proposed two hypotheses to investigate the potential mediating role of WtoFC in the relationship between workload and job satisfaction and between workload and family satisfaction. As anticipated (H6 partially supported), the WtoFC of both parents was found to mediate the link between their workload and job satisfaction. Both mothers’ and fathers’ workload indirectly and negatively impacted their job satisfaction through increased WtoFC. These findings align with previous research (Hong et al., 2021; Baş and Güney, 2022, p. 354). Our findings in Hypothesis 1a indicate no direct relationship between workload and job satisfaction in parents. Therefore, the mediating role of WtoFC implies that job satisfaction will likely diminish when parents experience higher workloads that contribute to WtoFC. These findings suggest that job satisfaction remains unaffected if the workload does not lead to WtoFC, as Kim et al. (2023) observed when studying the direct and indirect associations between workload, job stress, and WtoFC.

Additionally, we discovered that fathers’ WtoFC is a mediator between their workload and mothers’ job satisfaction, indicating that their workload also negatively influences mothers’ job satisfaction through their increased WtoFC. This insight into the interindividual mediating role represents a new contribution to the field.

We anticipated that WtoFC would mediate between the workloads of mothers and fathers and the family satisfaction of the three family members (H7). This hypothesis was partially confirmed by five significant mediating roles, one at an individual level and the others at an interindividual level. The first role demonstrated that fathers’ workload negatively impacted their family satisfaction indirectly by increasing their WtoFC. The other two show similar effects; that is, mothers’ workload was indirectly related to a decrease in fathers’ family satisfaction via an increment in mothers’ WtoFC, while also the fathers’ workload was indirectly and negatively related to the mothers’ family satisfaction by an increased WtoFC in the fathers. These findings align with previous results (Zhang et al., 2024). However, a different mediating role involved an indirect and negative association between fathers’ workload and the mothers’ family satisfaction, but in this case, via an increased WtoFC in mothers. Furthermore, the last mediating role expands the knowledge, showing that WtoFC can mediate between parents’ workload and family satisfaction. WtoFC may also mediate between one parent’s workload and the teenagers’ family satisfaction, as was obtained from the father’s workload to the adolescents’ family satisfaction.

It is crucial to underscore the findings related to fathers and mothers as they shed light on each parent’s unique challenges. Fathers experienced only one negative indirect impact on their job satisfaction, stemming from their workload. In contrast, mothers faced two adverse indirect impacts on their job satisfaction, from their workload and their partners’ workload through crossover. Despite these differences, fathers and mothers did not show variations in their job satisfaction scores, suggesting that other unexplored factors may explain the lack of differences. Additionally, both fathers and mothers experienced two adverse indirect impacts on their family satisfaction, from their workload and their partner’s workload through crossover. Notably, mothers also faced a direct negative impact from their workload, which could potentially account for their lower family satisfaction scores compared to fathers. These findings comprehensively understand the complex dynamics in parents’ family and work lives.

The results have significant consequences for comprehending the intricacies of family dynamics. Adolescents were influenced by two direct interindividual effects stemming from the workload of their fathers and mothers. Additionally, they experienced an indirect interindividual effect resulting from their fathers’ workload. It was observed that fathers’ workload indirectly and adversely affected the family satisfaction of adolescents through the fathers’ WtoFC. This finding indicates that fathers’ workload may hurt their parental performance, potentially leading to adopting a less assertive communication style and the deterioration of the father-child relationship. Thus, consistent with previous research (Frone et al., 1992), the interference of work in fathers’ lives dealing with the demands of adolescents within the family domain contributes to reduced family satisfaction. These implications underscore the need for a more nuanced understanding of the factors that influence family dynamics.

The findings of Hypotheses 6 and 7 highlight the importance of the fathers’ workload and WtoFC in affecting both the parents’ job satisfaction and the three family members’ family satisfaction. In parallel, these findings support the domain specificity model more than the source attribution perspective.

Nevertheless, comparing the domain specificity model (WtoFC → family satisfaction) and the source attribution perspective (WtoFC → job satisfaction) showed no statistical differences (H8 not supported). Hence, these results contradict studies that support the source attribution perspective over the domain specificity model (e.g., Yucel and Latshaw, 2020; Amstad et al., 2011; Shockley and Singla, 2011; Xu et al., 2018; Michel et al., 2011) and a previous study in Chile showing the opposite trend (Schnettler et al., 2024a). Further research is needed to understand better the lack of statistical differences between both approaches, even when comparing significant paths with non-significant paths (i.e., Mothers’ WtoFC → Father’s OJSS – Mothers’ WtoFC→ Fathers’ SWFaL, Fathers’ WtoFC → Adolescents’ SWFaL) – (Mothers’ WtoFC→ Adolescents’ SWFaL). However, one possible explanation may be associated with the low strength of all significant paths. At the same time, it is also plausible that these results may be related to the study of both approaches, taking three family members and two domains as outcomes in parallel.

In summary, our main findings comprise the direct negative effects of parents’ workload on adolescents’ family satisfaction and the different gender patterns involving indirect effects via WtoFC. The mothers’ workload indirectly and negatively affects their job and family satisfaction and the fathers’ family satisfaction. The fathers’ workload negatively and indirectly affects their and the mothers’ job satisfaction and the three family members’ family satisfaction. Another significant gender-related result is the direct effect between fathers’ WtoFC and the adolescents’ family satisfaction. Lastly, it should be highlighted that the comparison between the domain specificity model and the source attribution perspective shows that both were similar.

### 4.4 Limitations

Future research endeavors should address the constraints encountered in this study. It is imperative to recognize that the effects identified by the Actor-Partner Interdependence Model should not be viewed as causal relationships due to the cross-sectional nature of this study, thereby rendering causal verification unattainable. Consequently, there is a vital need for longitudinal, experimental, or quasi-experimental studies to establish causality. Moreover, the non-probabilistic nature of the sample employed in this study hinders the generalizability of the findings to the broader population of dual-earning parents with adolescent children in Chile, as it exclusively represented families with adolescents aged 10–15. Hence, future investigations must employ a probabilistic sampling approach to facilitate the applicability of the results to a broader populace and to examine the influence of parental workload on the well-being of older adolescents. Furthermore, as this study was confined to one city within a developing country in Latin America that adheres to traditional family structures, there is an inherent requirement for cross-cultural studies encompassing nations or cultures characterized by varying levels of gender equity and economic development. Additionally, while the questionnaire inquired about the workplace locations of the mothers and fathers, it failed to encompass inquiries relating to their working conditions. The questionnaire also omitted inquiries concerning adolescents’ time with each parent, their perceptions of the parent–child relationship, parental communication style, and external support (e.g., family or community support networks), which may moderate the results obtained in this study. To deepen our understanding of the relationships examined, future research must actively investigate the moderating effects of these variables across diverse scenarios. This approach will provide valuable insights that can significantly enhance the findings.

## 5 Conclusion

Our research offers fresh perspectives on the literature regarding the interaction between work and family, demonstrating how parental workload mainly affected their job and family satisfaction and one adolescent child’s family satisfaction indirectly by increasing both parents’ WtoFC. Gender differences should be highlighted. Considering the direct effects, it seems more harmful mothers’ workload; whereas mothers’ workload directly and negatively affected their and their children’s family satisfaction, fathers’ workload only directly and negatively affected the adolescents’ family satisfaction. By contrast, considering the indirect effects of parents’ workload via their WtoFC, it seems more detrimental to the fathers’ workload. Whereas mothers’ workload indirectly affected their job and family satisfaction and the fathers’ job satisfaction, fathers’ workload affected their and mothers’ job and family satisfaction, and adolescents’ family satisfaction.

A symmetrical crossover effect was identified among parents, wherein mothers’ WtoFC impacted fathers, leading to a decline in their family satisfaction, and vice versa. This reflects the mutual transmission of stress between parents, reducing family satisfaction for both. Furthermore, an asymmetric crossover effect was detected, indicating that only fathers’ WtoFC influenced mothers’ job satisfaction without a corresponding impact in the reverse direction. Additionally, fathers’ WtoFC extended to adolescents, resulting in diminished family satisfaction. These outcomes contribute to understanding the crossover effects from parents’ WtoFC to their adolescent offspring.

Examining the direct and indirect effects of parental workload, work-to-family conflict, job satisfaction, and family satisfaction on dual-earning parents and their adolescent children concurrently contributes to theoretical understanding. This research demonstrates the coexistence of the source attribution perspective and the domain specificity model within these family dynamics, encompassing individual and interpersonal effects that impact job and family satisfaction. According to the domain specificity model and the source attribution perspective, WtoFC theoretically mediates between the work and family domains at the individual level (Frone et al., 1992; Shockley and Singla, 2011). Our research findings have significant implications, as they expand the theory to demonstrate that WtoFC conflict serves as a mediator at the interindividual level, not only between partners in a couple but also from parents to their adolescent children.

It should also be noted that the interindividual mediating role of WtoFC was observed not only between the work and family domains but also between job demands and outcomes. Additionally, our results reveal gender differences, suggesting that the interindividual mediating role of WtoFC cannot be generalized for mothers and fathers.

This study also holds implications for further research. Future research should investigate the potential for WtoFC and psychological distress to serve as sequential mediators in the relationship between workload or other job demands and satisfaction levels experienced in family and professional domains. Subsequent investigations should evaluate the circumstances under the source attribution perspective and ascertain whether the domain specificity model holds precedence over the former or fails to demonstrate statistically significant variances, as evidenced in the present study. However, a critical area for prospective research inquiries involves comprehending the circumstances under which mothers can segregate their work-related stressors from their familial domain. This critical aspect warrants in-depth exploration. Analogous familial dynamics should be appraised when other occupational demands are implicated. Future research should also focus on the role of sibling support, as older siblings can play a crucial role in assisting parents with the care of younger siblings. This support has the potential to alleviate some of the demands placed on parents, thereby enhancing family dynamics, particularly in different family structures, such as single-parent families, non-traditional families, and divorced parents. Furthermore, variables such as family socioeconomic status, adolescent ages, number of children, parental employment types, and maternal weekly working hours influenced the outcomes as control measures; hence, future research needs to examine how these factors might moderate the study results to gain a deeper understanding of their impact. Moreover, future studies could adopt a qualitative approach to deepen our understanding of family dynamics. Techniques such as in-depth interviews or case studies could supplement quantitative results, offering richer insights into how parents and adolescents perceive and navigate work–family conflict daily.

From a practical standpoint, the comprehensive impact of parental workload on familial and professional satisfaction and its effects on the familial satisfaction of adolescent children necessitates the formulation of organizational strategies. These initiatives should be designed to align task allocation with prescribed working hours, thereby mitigating undue workloads. In addition, organizations ought to hire an adequate number of staff and distribute responsibilities equitably among them. It is also important for employers to understand their employees’ strengths and refrain from assigning tasks that exceed their capabilities, which helps avoid feelings of inadequacy. Furthermore, companies should offer comprehensive vocational training during the hiring process and afterward establish a clear division of labor and enable current employees to share their knowledge and experiences with newcomers. Organizations should also offer employees sufficient opportunities to manage the time allocated to different job responsibilities. Implementing such measures facilitates a more favorable professional milieu and engenders augmented job satisfaction levels among employees and their families within the familial sphere. These initiatives play a crucial role for countries embarking on reductions in weekly working hours, such as Chile, and for nations with anticipated amendments to their labor legislation shortly.

## Data Availability

The original contributions presented in the study are included in the article/supplementary material, further inquiries can be directed to the corresponding author.
